# "My Cancer Is Worth Only Fifteen Weeks"? A Critical Analysis of the Lived Experiences of Financial Toxicity and Cancer in Canada

**DOI:** 10.34172/ijhpm.2021.83

**Published:** 2021-08-15

**Authors:** Ambreen Sayani, Jessica Dilney, Janet L. Kuhnke, Tom McNeil

**Affiliations:** ^1^Women’s College Research Institute, Women’s College Hospital, Toronto, ON, Canada.; ^2^Nova Scotia Health Authority, Cape Breton Regional Hospital, Sydney, NS, Canada.; ^3^School of Professional Studies, Cape Breton University, Sydney, NS, Canada.; ^4^Cape Breton Cancer Centre, Sydney, NS, Canada.

**Keywords:** Canada, Cancer, Income Security, Financial Toxicity, Social Benefits, Income Replacement

## Abstract

**Background:** Cancer patients experience financial hardship due to rising expenses related to cancer treatment and declining income levels associated with reduced employability. Employment Insurance Sick Benefits (EI-SB) is a social income support program which provides temporary income replacement to Canadians when they fall ill. Although EI-SB is designed to maintain continuity of income during an illness, little is known about the perspectives of cancer patients who receive EI-SB. This knowledge can inform the development of public policies which are responsive to the needs and priorities of cancer patients.

**Methods:** We conducted a theory-informed thematic analysis of data collected from twenty semi-structured interviews with participants who were receiving care in a cancer centre in Cape Breton, Nova Scotia and had received EI-SB. A coding framework was developed using Taplin and colleagues’ intermediate outcomes of patient care across the cancer care continuum. Interpretation of findings was guided by the synergies of oppression theoretical lens.

**Results:** Three overarching themes describe the experiences of cancer patients receiving social income support: Economic exclusion, in which the structure of the labour market and social welfare system determine access to workplace benefits and continuity of reasonable income; financial toxicity, a vicious cycle of financial burden and increasing financial distress; and constrained choices, where cancer influences employability and lowered income influences the need to be employed.

**Conclusion:** Cancer patients need income support programs that are tailored to match their healthcare priorities. In addition, policies which strengthen working conditions and facilitate a reintegration to work when possible will be important in addressing the structural drivers of income insecurity experienced by cancer patients.

## Background

**Key Messages**

** Implications for policy makers**

- One in every two Canadians will face a diagnosis of cancer over the course of their lifetime. For cancer patients who are employed this can cause a disruption to their ability to earn income and subsequently cancer patients will resort to workplace benefits or income replacement programs offered through social welfare to meet their financial needs.
- Individuals employed in precarious working conditions, ie, temporary or contractually based employment with little or no benefits are more likely to report financial hardship and difficulty in returning to work after being diagnosed with cancer.
- Cancer patients who depend on publicly-funded income replacement programs such as Employment Insurance Sick Benefits (EI-SB) and Canada Pension Plan Disability (CPP-DB) find the programs ill-suited to the chronicity of cancer and the complexity of cancer treatment resulting in a perpetual struggle to make ends meet.
- Upstream public policies which strengthen working (and return to work) conditions must be combined with a redesign of both EI-SB and CPP-DB to match the needs of cancer patients. This response is needed in order to prevent the economic exclusion of cancer patients, enhance their quality of life and support their recovery.

** Implications for the public**

In Canada, income replacement programs provide financial compensation to individuals when they need to take time off work due to cancer. These programs are a patchwork of private programs (provided as workplace benefits) and public programs (provided through social services). Individuals working in temporary positions often do not have access to any workplace benefits and must rely upon income replacement programs such as Employment Insurance Sick Benefits (EI-SB) and Canada Pension Plan Disability Benefits (CPP-DB) in order to make ends meet. The duration and amount of income offered through these programs do not match the chronicity of cancer and the complexity of cancer treatment leaving cancer patients in financial hardship. Strong workplace benefits, an ability to return to decent work after cancer, and a revision of the EI-SB and CPP-DB benefits will be necessary in order to provide cancer patients with the support needed to recover from cancer and experience a good quality of life.

Statistically, 1 in every 2 Canadians can expect to face a diagnosis of cancer over their lifetime.^1^ With a 5-year survival for all cancers combined at 67%,^2^ approximately 2.4% of the Canadian population is currently alive with a diagnosis of cancer.^3^ Survivorship is associated with significant financial adversity as cancer patients frequently find themselves trapped between rising expenses related to cancer-care^4-11^ on one hand and reduced employability with declining levels of personal income^12-14^ on the other hand. Being unemployed or having a low income is associated with poorer cancer adjustment, increased health problems, reduced quality of life, and earlier mortality.^4,5,8,15,16^ For cancer patients, financial stability is an important part of maintaining living conditions, dignity and self-respect.^17^

 On average cancer patients report taking 151 days of sick leave following their diagnosis and up to a third are unable to return to work.^18^ The ability to return to work is dependent on the age at diagnosis, site of cancer, intensity of treatment, frequency of physical symptoms, or flexible employment and availability of (re)training services.^18,19^ Return to work is also directly related to significantly high out-of-pocket costs related to medical care and non-medical expenses^4-7^ which can lead to a balancing act where patients may choose to skip medications or prioritise returning to work over receiving treatment.^20^ This financial burden of cancer is amplified at the intersections of marginalizing social identities such that gender, race and access to resources such as housing and employment determine the degree of vulnerability experienced resulting in higher mortality and poorer quality of life for individuals living with social disadvantage.^21-24^

 In Canada, a patchwork of income replacement programs are in place to support individuals when they are required to take time off work due to an illness (Table 1). Less than half of all Canadians are supported with workplace benefits which can provide adequate income coverage^25^ and subsequently most individuals will depend on publicly funded programs such as Employment Insurance Sick Benefits (EI-SB) for financial support when they fall ill. EI-SB provides significantly less income and is means-tested so individuals must prove eligibility by meeting certain criteria. To receive EI-SB, cancer patients must have accumulated more than 600 insured hours over a 52 week employment period and can expect to receive up-to a maximum of 55% of their regular work earnings for a duration of 15 weeks.^26^ Whilst it is understood that cancer patients experience financial hardship and reduced employability, little is currently known about the perspectives of patients who depend on EI-SB for income support. Understanding these lived experiences can illuminate the needs and priorities of cancer patients and inform the development of cancer-patient responsive public policies.

**Table 1:** Summary of Canadian Income Support Programs

**Benefit**	**Who Is Eligible?**	**What Is Covered?**	**Notes**
EI-SB (Government of Canada program)	Paid EI premiums. Unable to work due to a medical reason. Regular weekly earnings must have decreased by more than 40%. Have accumulated 600 insured hours in the last 52 weeks.	55% of work earnings for 15 weeks.	
CPP-DB (Government of Canada program)	Must have contributed to CPP in 4 of the last 6 years; or 3 of the last 6 years if you have contributed for at least 25 years. Medical condition must be considered both severe and prolonged. Severe refers to being incapable of regularly pursuing any substantially gainful occupation. Prolonged refers to the illness being long continued and of indefinite duration or is likely to result in death. Have to be between the ages of 18 and 64.	Pays flat rate of $485.20 (2018) plus 75% of a person’s calculated CPP retirement pension. Maximum monthly benefit (2020) $1175.83; average monthly benefit (2020) $672.87.	
Short-term Disability	Income replacement offered through the employer usually 9-52 weeks; average length 6 months.	55%-70% of work income.	
Long-term disability	Income replacement insurance offered through employer 2-3 years or longer, possibly until age 65.	60%-70% of work income.	Deducted dollar for dollar by Long-term disability if also approved for CPP-DB.
EI regular benefits (Government of Canada program)	Income replacement for individuals who have lost their jobs through no fault of their own (but not because of sickness). Must have been employed in insurable employment; have been without work and without pay for at least seven consecutive days in the last 52 weeks; have worked for the required number of insurable employment hours in the last 52 weeks or since the start of last EI claim, whichever is shorter.	55% of average insurable earnings, up to a maximum amount weekly ($573/wk for 2020). Eligible to receive from 14 weeks to a maximum of 45 weeks.	People off work due to illness not eligible.

Abbreviations: CPP, Canada Pension Plan; EI-SB, Employment Insurance sickness benefits; CPP-DB, Canada Pension Plan Disability Benefits; EI, Employment Insurance.

## Methods

###  Study Design and Theoretical Approach

 Research ethics board approval was obtained from Nova Scotia Health Authority Research Ethics Board. We used theoretical thematic analysis as a way to conceptualise the research questions, collect the study data, and interpret our findings^27^ using the synergies of oppression analytical lens.^28^ The synergies of oppression is a theoretical lens which conceptually locates the intersections of social identity (such as gender, age and disability) with the social determinants of health (such as income and education) and social geography (such as service accessibility and urban or rural location). This theoretical tool has been previously applied to conceptualise the structural inequalities which underpin inequities in breast cancer care,^23^ genetic testing for heredity breast cancer,^29^ access to lung cancer screening^30^ the development of national cancer control policies,^31^ and can inform the interpretation of patient perspectives on EI-SB based on social location. We coded our data using a framework developed from Taplin and colleagues^32^ intermediate outcomes of patient care across the cancer care continuum. These indicators include the stage of cancer diagnosis, quality of life, quality of death and financial burden.^32^

 Our study questions were:

1. What is the cancer patients experience of receiving EI-SB?
2. What is the impact on quality of life for cancer patients who depend on income support programs such as EI-SB?
3. How can cancer patients be better financially supported?

###  Participant Recruitment and Setting

 Participants were individuals who had been diagnosed with cancer and had received social income support. Participants were recruited to the study through one of the following ways: (*i*) potential participants were informed of the study by a social worker within the patients circle of care and handed a study poster; (*ii*) posters were placed in the waiting area of the social services department in the cancer care centre under the Nova Scotia Health Authority; (*iii*) information about the study was shared through radio and newspaper advertising. Interested participants called a telephone number (directed to TM) that was shared through the posters and media. During this phone call, eligible participants agreed on a date and time for the interview. Participants were included if they were patients of the Cape Breton Cancer Centre and had received EI-SB. Participants were not excluded if they were or had ever received additional income support including Canada Pension Plan Disability Benefits (CPP-DB), short or long-term disability. Participants were informed of the purpose of the interview, approximate duration, and potential harms and benefits of participation. Participants who agreed to take part in the study provided written consent before interviewing began.

###  Data Collection

 Data were collected through semi-structured interviews conducted face-to-face. The interview guide was developed by one social worker of the cancer centre, and reviewed by another social worker. During the interviews, participants were asked about their cancer diagnosis, treatment and side-effects. In addition, participants were asked about their current sources of income and their experiences with EI-SB. All interviews were conducted by 2 social workers who are part of the research team (JD and TM). Interviewing continued until no new themes emerged and conceptual saturation^33^ was reached after interviews with twenty participants. Interviews were audio-recorded with participants permission and transcribed verbatim.^34^ To ensure validity transcripts were reread. Field notes taken during the interviews were added to the transcribed files. All data was entered into a qualitative software program (NVivo version 12) for data management.

###  Data Analysis

**Figure 1:**
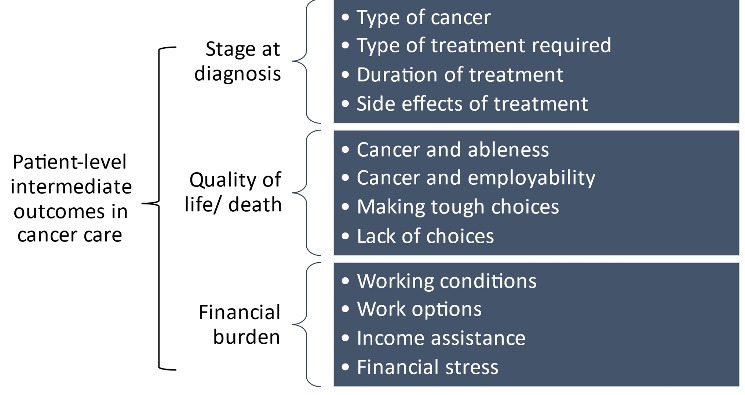
Coding Tree Based on Taplin and Colleagues’ Intermediate Indicators of Patient Outcomes in Cancer Care and Sample Codes.

 All authors read through the transcripts individually, and subsequently met to identify initial codes and create an overarching coding framework based on Taplin and colleagues’ intermediate indicators of patient outcomes in cancer care.^32^ Subsequently, line-by-line coding was applied to the texts. Additional codes were developed and modified through group peer discussion until all lines of text had been coded. Figure 1 shows the coding tree framework and sample codes. The multidisciplinary nature of the research team enhanced reflexivity^35^ allowing us to code any data which did not match our theoretical inquiry. AS is a medical doctor and critical qualitative researcher on health inequities. JD and TM are social workers in a cancer care centre and clinical researchers. JK is a registered nurse and a healthcare researcher. We identified our final 3 themes through iterative discussions between the research team.

## Results

###  Characteristics of the Study Sample

 We interviewed 20 participants (14 female, and 6 male) who had been diagnosed with cancer and had received EI-SB. Two participants were below the age of 51 years; 16 participants were between 51-64 years of age; and 2 participants were over the age of 65 years. All study participants were ethnically white Caucasian. Seven participants recounted their cancers as early stage (stage 1 or 2), 5 participants reported advanced cancer (stage 3) and 8 participants described their cancer as metastatic (stage 4). Participants also recounted receiving a patchwork of income support through Canada Pension Plan (CPP), CPP-DB, Regular Employment Insurance (EI), Income Assistance through welfare, and/ or the Cape Breton Cancer Centre Patient Fund. The Cape Breton Cancer Centre Patient Care Fund is a charity established in 2005 at the Cape Breton Cancer Centre in conjunction with the hospital foundation of the Cape Breton Regional Hospital. It is funded by community donors. It financially aids patients of the Cape Breton Cancer Centre with expenses that have resulted from a cancer diagnosis. These expenses include, but are not limited to, medical travel and lodging, medical equipment, private homecare, prescriptions and household expenses. Participant characteristics are described in Table 2.

**Table 2:** Participant Characteristics

**Participant ID**	**Age**	**Gender**	**Ethnicity**	**Cancer Site**	**Cancer Stage**	**NS-SEC** ^a^ **Occupational Class Prior to Cancer Diagnosis**	**Employment Status at Time of Interview**	**Workplace Benefits**	**Benefits Accessed (Y = Yes; N= No) and STATUS (Amount/Month; or Eligibility)**								**Time in Weeks Without Any Income**
									**EI-SB**	**CPP-DB**	**CPP**	**NS-IA**	**EI**	**Short-term Disability**	**Long-term Disability**	**Cape Breton Cancer Fund**	
PP1	51-64	Female	White	Breast	1	5	Employed	Drug plan	Y	Denied	Ineligible	Denied	N	N	N	Y	32
PP2	<50	Female	White	Breast	4	5	Unemployed	None	Y	Ineligible	Ineligible	Ineligible	N	N	N	Y	0 income after 15 wks. of EI-SB terminated
PP3	51-64	Female	White	Breast	1	1	Employed	Drug plan	Y	N	N	Ineligible	N	N	N	N	28
PP4	51-64	Male	White	Prostate	3	5	Unemployed	None	Y	Y	Ineligible	Ineligible	Y	N	N	Y	8
PP5	51-64	Male	White	Pancreas	4	5	Unemployed	None	Y	700/month	Ineligible	Y	N	N	N	Y	4
PP6	51-64	Female	White	Breast	2	4	Unemployed	Drug plan; LTD	Y	N	Ineligible	N	N	N	Y	N	8
PP7	51-64	Male	White	Rectal	3	5	Unemployed	None	Y	Applied	Ineligible	Ineligible	N	N	N	Y	2
PP8	51-64	Female	White	Breast	1	4	Unemployed	None	Y	619/month	Ineligible	Y	N	N	N	Y	6
PP9	51-64	Female	White	Breast	4	5	Unemployed	None	Y	800/month	Ineligible	Ineligible	N	N	N	N	0
PP10	51-64	Male	White	Rectal	4	5	Unemployed	None	Y	Ineligible (at the time)^b^	282/month	Ineligible	N	N	N	Y	0
PP11	51-64	Female	White	Breast	1	4	Employed PT	None	Y	700/month	N	N	N	N	N	Y	8
PP12	<50	Female	White	Breast	4	5	Employed PT	None	Y	650/month	Ineligible	Ineligible	N	N	N	Y	0
PP13	>65	Female	White	Breast	3	5	Unemployed	None	Y	Ineligible	800/month	Ineligible	N	N	N	Y	0
PP14	51-64	Male	White	Rectal	3	5	Unemployed	None	Y	Applied	Ineligible	Ineligible	N	N	N	Y	0
PP15	51-64	Female	White	Breast	1	5	Unemployed	None	Y	Ineligible (at the time)^b^	320/month	N	N	N	N	N	24
PP16	51-64	Female	White	Breast	1	5	Unemployed	None	Y	Applied	Ineligible	Ineligible	N	N	N	N	0
PP17	51-64	Female	White	Lung	4	5	Unemployed	None	Y	646/month	Ineligible	Ineligible	N	N	N	Y	3
PP18	51-64	Male	White	Lung	4	5	Unemployed	None	Y	Ineligible (at the time)^b^	Y	Ineligible	N	N	N	Y	0
PP19	51-64	Female	White	Breast	3	4	Employed	None	Y	N	Ineligible	Ineligible	N	N	N	N	0
PP20	>65	Female	White	Colon	4	4	Unemployed	None	Y	Y	Ineligible	Y	N	N	N	N	0

Abbreviations: LTD, Long-term disability; EI, employment insurance; EI-SB, employment insurance – sickness benefits; CPP, Canada Pension Plan; CPP-DB, Canada Pension Plan Disability Benefits. ^a^NS-SEC is the National Statistics Socio-Economic Classification which categorises occupations into five social groups: Class 1, higher managerial, administrative and professional occupations; Class 2, intermediate occupations; Class 3, small employers and own account workers; Class 4, lower supervisory and technical occupations; Class 5, semi-routine and routine occupations including manual labour. ^b^As of January 1, 2019 persons in receipt of CPP regular benefits could apply for CPP-DB.

###  Themes

**Figure 2:**
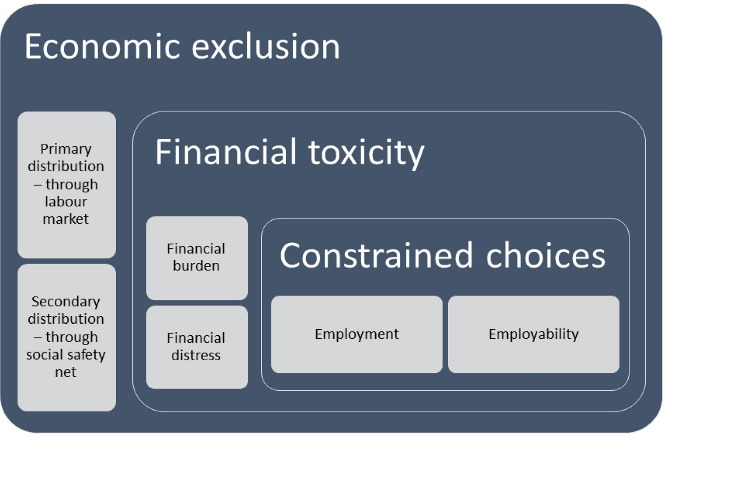
Themes and Subthemes of the Study.

Figure 2 shows the main themes and subthemes of our study: Economic exclusion, financial toxicity and constrained choices. The findings are discussed in detail below:

####  Economic Exclusion

 Economic exclusion is described as the systematic and structural economic divide between individuals which occur as a result of labour market policies and social welfare systems that fail to fairly distribute and redistribute economic assets in society.^36,37^

 In our study, participants described how their economic stability and access to benefits was determined by their employment status prior to being diagnosed with cancer. Most participants recounted being employed in precarious working conditions with little or no access to any health plans or income support as described by this participant:

*“There was no plan through my work. I work for a company that unless you work 30 hours a week you’re not going to be on their medical plan, and most places you work for today, you know, you are working the minimum wage job, you know? They are going to keep you under those 30 hours because they don’t want you to be on the plan so you’re not going to get the benefits” *(PP16).

 Being employed in a contract job also implied that participants lost their jobs as a result of their cancer diagnosis as recounted by this participant:

*“I was in a contract, so I had to walk away from the contract when I got diagnosed” *(PP19).

 Subsequent to cancer, all participants experienced unemployment. All participants had applied for and received EI-SB. Most participants recounted the income amount received through EI-SB as a bare minimum as recounted below:

*“If all we are giving them is bare bones minimum income, and that’s really what EI is” *(PP19).

 Participants described the challenge of meeting their daily needs when income had been halved through EI-SB. This participant recounted the irony of receiving half of their regular income, when needs and expenses could not in turn be halved:

*“If you take a person that’s making $500 a week and then all of a sudden they are forced into this situation (receiving EI-SB) and they’re getting half of that (amount), well, you can’t go to your local grocery store and they’re going to charge you half your grocery bill because of your situation. So, you know, and that pertains to everything you do. They’re not going to start cutting things in half for you” *(PP10).

 Participants described the duration of EI-SB (15 weeks maximum) as a complete mismatch with a chronic illness such as cancer for which treatment was complicated and lengthy:

*“Like you know for people who are diagnosed with cancer, if they can figured out a way to help people or cure people with cancer in 15 weeks then I am all for it but there is no way when you are diagnosed with cancer that you can survive on 15 weeks of EI” *(PP11).

 Participants described the 15 week duration of EI-SB as short and insufficient. For participants with advanced stage cancer this was particularly arduous. One participant with terminal cancer drew parallels between the duration of EI-SB (15 weeks) and regular EI benefits (1 year) for which they were ineligible:

*“It seemed so short at the time (15 weeks), and then all of the sudden, it was finished. I was in the middle of dying from cancer for God sake. You know, if you lose your job, you have a year, but if you have cancer you have 15 weeks. I don’t understand that” *(PP20).

 Participants used words such as, “slapped,” “kicked” and “worth” to describe their frustration at not being able to tap into regular EI after having paid into it throughout the period of their employment:

*“When you worked all of these years why can’t you collect regular EI with it? It does not make sense. There are all of those years that I worked. Why? To get slapped in the face with $650 a month” *(PP12).

*“After paying into the EI for so many years you feel like you are just kicked while you are down” *(PP11).

*“My cancer is only worth 15 weeks?” *(PP2).

 After termination of EI-SB, participants described their struggle to prove eligibility for other income support programs. For CPP-DB, participants with early stage cancer were not considered eligible. This participant was denied CPP-DB twice as he could not prove that his cancer was severe enough:

*“I applied for Canada Pension and I was denied twice. Cancers (are) not considered severe or prolonged. But, cancer can kill you! But, (they say) it’s not severe! So that’s iffy on my part” *(PP1).

####  Financial Toxicity

 Financial toxicity is described as the objective (financial burden) and subjective (financial distress) experience of economic strain.^9^

 Several participants recounted how financially coping with aspects of their daily life became entangled with cancer. This included the cost of meeting regular monthly expenses as recounted by this participant:

*“You still have all of your bills to pay. Life doesn’t stop because you get cancer” *(PP1).

 For many participants this entanglement resulted in financial hardship and a struggle to make ends meet. Participants recounted how they prioritized expenses between medicines, food or gas:

*“If it was medical, it came first. If it was food it came second. Gas came third. Anything after that we ain’t doing it” *(PP1).

 Participants recounted gnawing away at any savings in order to make ends meet until there was nothing left as described by this participant:

*“I didn’t have a lot of savings. But you’re continually chewing away at that. And it just comes to a point that the well dries up after a while” *(PP10).

 Participants who were unable to return to work found themselves in dire financial constraints. One participant described how she struggled to prove eligibility for welfare support whilst facing her own rapidly declining health:

*“You spend so much time you are proving you are poor, getting photo copies of things … You have no idea how time consuming it is to be poor … But (even then) I was still below the poverty. Plus I was dealing with the fact that I was dying and my daughter was going to be an orphan. It was so terrible” *(PP20).

 The same participant suggested that a General Annual Income (which is not means-tested) would be a good way to provide income security to cancer patients in a way that would allow them to make ends meet without relying on a patchwork of supports:

*“They have to get the cancer patients out of the welfare roles. They have to make accommodation for people who have worked all their lives and hit a hard patch and have nowhere to turn. The basic annual income, I’m telling you, if I had that, I would have never had to go to welfare or CPP because I would have been covered. I would have been able to pay my car insurance, house insurance, mortgage and buy some half decent food. You know instead, I had all this piece meal stuff … donations, friends*…*it shouldn’t be this way, it’s wrong” *(PP20).

####  Constrained Choices

 We build on Max Weber’s^38^ concept of life conduct and the limitation of one’s choices to describe constrained choices as the variety of opportunities available to individuals based on access to material resources and social structure.

 As a result of a significantly reduced income, some participants had no option but to return to the workforce. Participants who were employed at the time of the interview recounted this as a lack of choice as described by this participant:

*“I could not financially stay off work. It was not an option for me to not earn whatever income I could” *(PP19).

 The need to return to work before being physically well enough to do so in order to afford basic expenses such as rent is recounted by this participant who was receiving income support which was not even sufficient to cover this monthly rent:

*“(I got) $700 a month. My rent is more than that. So I am forced. My options are, go back to work. I am a workaholic anyway, but am I going to go back sick? I got no choice; I got to go back to work” *(PP5).

 Several participants recounted health challenges that limited their ability to return to full time employment. For participants receiving chemotherapy this implied avoiding a crowded workplace where they would have to compromise their health by exposing themselves to a higher infection rate as described by this participant:

*“And when your immune system is compromised, like to throw me back into my workplace, there is, somebody’s sick all the time like that would be compromising me until I’m healthy enough to return there” *(PP6).

 Other participants were not able to return to their prior employment because of the physical impact of cancer on their health. One participant recounted how she felt weak and tired most of the time, and despite her desire to return to work she was not physically able to do so:

*“Right now, I’m very weak. My legs are tired all the time. They are just like Jello. I’m walking with a cane*.* So it’s not … for the amount of walking that I do at the office, um, it’s kind of hard to walk with a cane and carry a bunch of papers or whatever. And plus the tiredness, I’m tired a lot so I wouldn’t be able to just go and say ‘I’m going to take a nap now,’ I’d have to work around … I’d be draining myself too much if I went back to work right at this moment, I’d love too, but, it just can’t be done” *(PP17).

 Several participants felt that their ability to perform well at a job had been compromised by the impact of cancer on their health and wellbeing as described by this participant:

*“Most jobs out there, people are looking for good, solid employees they can depend on. And, now due to my sickness, I don’t feel I’m one of them” *(PP10).

 Participants that were willing and able to work described themselves as trapped between a health condition requiring intensive monitoring and support on one hand, and a lack of flexible job opportunities that would be suitable to their needs on the other hand:

*“You have a double-whammy. You are trying to recover from your illness and you don’t have a job … It’s like (the lack of) job opportunities too, they are coinciding at the same time. Who is going to hire you if you’ve got doctor’s appointments that you’re going to back and forth. So it seems like you’re contending with kind of a struggle on both ends” *(PP3).

 Several participants shared a desire to return to work part time. As recounted by this participant, returning to work part time would have been the ideal case scenario, facilitating her to gradually reintegrate into the workforce:

*“In an ideal world I could have returned to work part time, but I didn’t have that option because I didn’t have a job to return to. So, reintegration into the workforce would have been better” *(PP19).

## Discussion

 In Canada, a patch work of short and long-term income supports are available through either public or private health plans to assist individuals when they are unable to return to work because of illness or disability. Private income support plans are benefits provided through employment. However, approximately half of all employed Canadians do not have adequate disability coverage through their workplace^25^ and this is directly linked to the increasing precarity of jobs which provide minimum wage and offer little or no workplace benefits.^25,39^ Individuals who do not have workplace benefits must resort to social insurance programs such as EI-SB for income support when they need to take time off work due to an illness.

 While the goal of EI-SB is to provide income continuity in the face of an illness, our study participants described how the short duration of EI-SB (15 weeks) and strict eligibility criteria for other income support programs such as CPP-DB (disease must be both severe and prolonged rendering individual incapable of working) left participants scrambling to make ends meet and with feelings of anger and frustration as they juggled their health and financial needs. Current income support programs are ill-suited to the complex needs of cancer patients for whom treatment can take between 16 to 24 weeks and include a combination of surgery with or without chemotherapy or radiotherapy. This duration does not include time needed for additional treatment (further surgery/radiation), side-effects of therapy, or the physical and psychological toll of cancer on the patient. Further to this, income replaced through EI-SB and CPP-DB is at levels below the poverty line. Participants in our study endured financial hardship due to their healthcare needs and concurrently struggled with finding suitable employment. Choices were most constrained for participants who were women and individuals between the ages of 51-64 years.

 The vicious cycle of financial burden and financial distress did not occur in a vacuum. Participants described how they were structurally excluded from participating in full-time, well-paid jobs with benefits. Indeed, the majority of our study participants were working contractual jobs with no benefits. Subsequently, when diagnosed with cancer, participants were forced to leave their jobs and had no financial recourse. The structure of the labour market is responsible for the primary distribution of material capital through the provision of adequate income and financial security.^40^ In Canada, contingent labour, ie, temporary workers with no benefits ^41^ make up approximately 30% of the labour market. As full-time, well-paying jobs continue to decline in Canada^42^ it is crucial to implement public policies which strengthen working conditions for employees. This is particularly important given the parallel increase in cancer incidence across the country.^1^ Once cancer patients are physically and psychologically ready to return to work, it will be important to support them through multidisciplinary programs which facilitate retraining and reintegration into the labour market. Ideally, these programs should be an integral component of cancer survivorship care.^43^

 The social welfare system is responsible for the secondary redistribution of material capital. Recent cuts in social services spending leading to reduced social assistance, and a means-tested distribution policy imply that many individuals are left scrambling to prove eligibility. All of our study participants had received EI-SB. However, only a few were eligible for CPP-DB. Others accessed other forms of income support such as CPP or Income Assistance through welfare. The Cape Breton Cancer Fund was an additional source of income support for participants who could not qualify for any of the publicly-funded income programs. Most participants experienced multiple weeks where they did not have any source of income support. The patchwork of income support programs resulted in stress, financial hardship, and loss of dignity. All of these are associated with deepening financial toxicity^9^ and poorer cancer-related health outcomes.^7,16^ Twelve of our participants had advanced stage cancer (stage 3 or 4). The average income support for these participants was reported at $500 a month, an amount well below the low income cut-offs in Canada.^44^ These participants recounted a continuous struggle with bills, and payment for food and gas.

 Most of our study participants were between the ages of 51-64 years. For this demographic age group, described as preseniors,^45^ financial hardship was intensified as participants found themselves caught between a loss of income due to illness, a lack of job opportunities suitable to their needs, and an inability to tap into pension and social benefits available to those over the age of 65 years. Participants described how they depleted any or all of their savings, struggled to prove their eligibility for other income support programs, and contested between diminishing health and the ability to work. Given that the incidence of cancer rapidly begins to increase after the age of 50 years,^2^ it is important that public policy programs take into consideration the complex challenges of cancer risk that intersect with a propensity towards economic hardship for preseniors.^45^

 Our study illuminates the urgent need to revisit the structure of income assistance programs for cancer patients. Among industrialized nations, Canada’s EI-SB program provides one of the shortest duration of benefits.^46^ Ninety countries provide benefits of a longer duration and 127 countries provide higher levels of wage replacement.^47^ For those unable to return to work, the CPP-DB provides less than the low income cut-off^44^ and is considered among the least generous of public initiatives.^39^ The duration and amount of benefits do not currently correspond with the complex treatment and prognosis of cancer. Alternate eligibility criteria for regular EI and/or CPP for patients with advanced stage cancer will ensure that they are able to avail benefits which they have accrued during their end-of-life care. As suggested by one participant, a Guaranteed Annual Income^48^ would alleviate much of the stress associated with proving eligibility, and would enable cancer patients to meet their monthly expenses while focusing on recovery or palliation in a dignified way. Ultimately, emphasis needs to be placed on the upstream determinants of cancer risk, treatment and survival^24^ in order to improve the quality and distribution of the social determinants of health as they intersect across the cancer care continuum.^23^

 Our study participants were all white, mostly between the ages of 51-64 years, majority female and part of the contingent labour workforce. This can reflect the way in which we recruited participants from social services at a cancer care centre with a demographically homogenous patient population. However, it can also illuminate the disproportionate representation of individuals who are preseniors in precarious working conditions that need income support in order to make ends meet when they are diagnosed with cancer. This warrants further exploration. The over-representation of females in our study sample mirrors what is currently known in the literature about gender-based inequities. Women are more likely to be precariously employed with little to no access to benefits^49^ and have fewer opportunities to return to work after cancer.^50^

 The increasing incidence of cancer with high levels of survivorship on one hand, and the greater economic exclusion of individuals through the labour market and social welfare programs on the other hand create a profound gap in income support available to cancer patients when they fall ill and are unable to return to work. Closing these gaps will require a mix of working conditions that provide financial security with workplace benefits along with a redesign of public income support programs to respond specifically to the chronicity and complexity of the needs of cancer patients. As a qualitative study, we do not expect our work to be generalizable, however we believe that by describing our study setting and participant population, our findings will be transferable to other jurisdictions seeking to address income support gaps for cancer patients.

## Conclusion

 Cancer patients employed in precarious working conditions can find themselves unemployed and without access to any workplace benefits upon falling ill. As a result, cancer patients turn to social welfare programs such as EI-SB for income support during their illness. Financial compensation awarded through the EI-SB is insufficient in duration. The CPP-DB has strict eligibility criteria which cancer patients can struggle to prove. The income offered through both programs is bare minimum and cancer patients, including those with terminal cancer can find themselves struggling to make ends meet as benefits offered are well below the poverty line. An increasing incidence of cancer, a rise in precarious working conditions, and cuts to social services spending result in a porous system which fails to protect and support cancer patients during a period of intense financial hardship. A redesign of income support programs tailored to the needs of cancer patients is urgently needed. In addition, public policies which strengthen working conditions, provide adequate income support to Canadians when they are ill, and facilitate a reintegration into work when possible will be important in responding to the complexity and chronicity of cancer.

## Acknowledgements

 The authors would like to thank the study participants who have contributed to our shared understanding of the lived experiences of income insecurity as a cancer patient.

## Ethical issues

 Research ethics board approval was obtained from Nova Scotia Health Authority Research Ethics Board.

## Competing interests

 AS reports a Postdoctoral Fellowship Award in Patient-Oriented Research from the Canadian Institutes for Health Research. JD, JLK, and TM have nothing to disclose.

## Authors’ contributions

 Study was conceptualised by TM. Material preparation was done by TM and reviewed by JD. Data was collected by TM and JD. Coding was done by AS, JLK and TM. AS, JLK, JD, and TM analysed the data. The first draft of the manuscript was written by AS and all authors commented on previous versions of the manuscript. All authors read and approved the final manuscript.
